# A Potential Pneumothorax Induced by Immune Checkpoint Inhibitors: A Case Report and Literature Review

**DOI:** 10.3390/medicina60101634

**Published:** 2024-10-06

**Authors:** Yoon-E Shin, Hyuk Kim, Jeong-Ju Yoo, Sang Gyune Kim, Young Seok Kim

**Affiliations:** Department of Internal Medicine, Soonchunhyang University Bucheon Hospital, Bucheon 14584, Republic of Korea; passion_97@naver.com (Y.-E.S.); qwester123457@gmail.com (H.K.); mcnulty@schmc.ac.kr (S.G.K.); liverkys@schmc.ac.kr (Y.S.K.)

**Keywords:** pneumothorax, immune-related adverse events, immune checkpoint inhibitor, atezolizumab, immunotherapy

## Abstract

*Background and Objectives:* Immune checkpoint inhibitors (ICIs), which target immune checkpoints in cancer cells, are increasingly used as a mainstay in anticancer treatment. The combination of atezolizumab and bevacizumab is also a first-line treatment for hepatocellular carcinoma (HCC). However, ICIs can cause immune-related adverse events (IrAEs) which range from mild to severe, potentially leading to the need for discontinuing immunotherapy. We report a case of a pneumothorax, a rare side effect caused by IrAEs. *Materials and Methods:* This paper reports a case of a 78-year-old male HCC patient who developed a recurrent pneumothorax, suspected to be an adverse effect of ICIs. *Results:* The patient was a current smoker with a 30 pack-year smoking history. Prior to initiating ICIs, a chest CT scan showed mild emphysema and fibrosis attributable to smoking. Following ICI treatment, the patient developed a recurrent pneumothorax. Further tests revealed no underlying cause for the pneumothorax other than the ICIs and smoking, and there were no signs of intrapulmonary metastasis or pneumonitis. *Conclusions:* When a pneumothorax occurs in a patient undergoing immunotherapy, it is important to consider it as a potential adverse effect of the treatment. Special attention should be given to the possibility that immunotherapy may exacerbate underlying lung conditions. Patients should be advised on the importance of smoking cessation. As there are currently no guidelines for resuming immunotherapy after a pneumothorax, it is crucial to weigh the risks and benefits and consider dose reduction or discontinuation of the medication.

## 1. Introduction

Immunotherapy works by enhancing the body’s immune system to target and combat cancer cells. Among these therapies, immune checkpoint inhibitors (ICIs) are a form of targeted therapy that disrupt the immune evasion mechanisms of cancer cells by inhibiting cytotoxic T lymphocyte-associated antigen 4 (CTLA-4) and the programmed cell death protein 1 pathway (PD-1/PD-L1). These treatments are known for their precision and typically have fewer side effects compared to traditional chemotherapies, which cause systemic toxicity. Consequently, ICIs have gained significant attention in cancer treatment. In hepatocellular carcinoma (HCC), the combination of atezolizumab and bevacizumab is currently used as first-line systemic therapy [1,2,3].

However, ICIs can cause immune-related adverse events (IrAEs) that differ from those of conventional chemotherapies. IrAEs can affect various organs, including the skin (rash), gastrointestinal tract, liver, endocrine system, lungs (pneumonitis), and heart (myocarditis) [4]. These side effects can range from mild to severe, potentially leading to the need to discontinue immunotherapy [5].

Respiratory side effects from ICIs often include pneumonitis, which can sometimes lead to a secondary pneumothorax. In rare cases, a sarcoid-like granulomatous reaction characterized by subpleural micronodular opacity and hilar lymphadenopathy may occur [6]. A pneumothorax is an uncommon side effect of the atezolizumab and bevacizumab combination. This paper reports a rare case of a 78-year-old male HCC patient who developed a recurrent pneumothorax suspected to be an adverse effect of ICIs. Additionally, we review the literature on pneumothorax cases associated with ICIs.

## 2. Case Presentation

A 78-year-old South Korean man undergoing atezolizumab and bevacizumab chemotherapy for HCC presented to the emergency room (ER) with shortness of breath and coughing persisting for 3 days. The symptoms began 3 days prior to presentation and were not accompanied by fever or sputum. In July 2018, he was diagnosed with alcoholic liver cirrhosis and HCC at a hospital in South Korea. The HCC was classified as Barcelona Clinic Liver Cancer (BCLC) stage C and modified Union for International Cancer Control (mUICC) stage III. The patient underwent a left lateral sectionectomy for a 5.5 × 4 cm mass in segments 2/3 and transhepatic arterial chemoembolization (TACE) for a residual mass in segment 4. He subsequently received imaging tests every 3–6 months to monitor for recurrence.

In January 2022, a computed tomography (CT) scan revealed HCC recurrence, with a 3.7 cm tumor in segments 5/8 and suspicious bile duct invasion. The patient’s liver function remained well preserved, with a Child–Pugh class A rating and a Model for End-Stage Liver Disease (MELD) score of 7. Opting against further surgical treatment, he began three cycles of TACE followed by radiotherapy. Despite this, follow-up CT scans showed tumor enlargement and disseminated bone metastases, with an elevated AFP level of 69.1. Consequently, ICIs with atezolizumab and bevacizumab were initiated in November 2023.

The patient was a current smoker with a 30 pack-year history and did not quit smoking during ICI therapy. He had no previous history of a pneumothorax. On physical examination, his vital signs were as follows: body temperature of 36.5 °C, blood pressure of 120/80 mmHg, heart rate of 80 beats per minute, and respiratory rate of 19 breaths per minute. Decreased breath sounds were noted on the left side. Laboratory tests for infection markers were within the normal range, with a white blood cell count of 7.91 × 10^3^/μL and a C-reactive protein level of 0.24 mg/dL.

**Figure 1:**
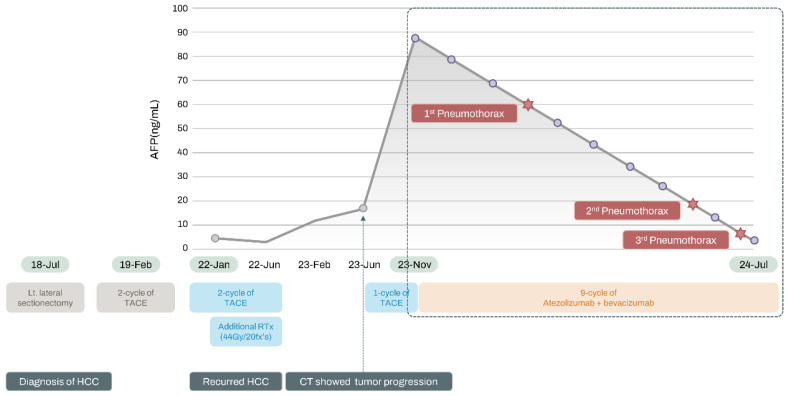
The clinical course of the patient. AFP: alpha-fetoprotein; CT: computed tomography; HCC: hepatocellular carcinoma; TACE: transcatheter arterial chemoembolization.

**Figure 2:**
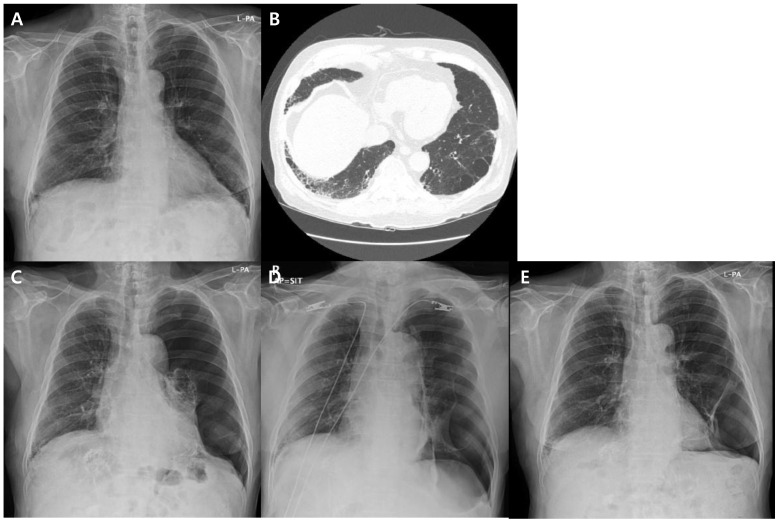
Radiologic findings of patient. (**A**,**B**) Before atezolizumab and bevacizumab treatment; (**C**) first pneumothorax; (**D**) second pneumothorax; (**E**) third pneumothorax.

The clinical course of the patient related to the pneumothorax is detailed in Figure 1. Prior to initiating ICIs, chest X-ray (Figure 2A) and CT (Figure 2B) scans revealed mild emphysema. After three cycles of atezolizumab and bevacizumab treatment, the patient presented to the ER with shortness of breath and coughing. A chest CT scan showed a pneumothorax in the left lung, and a tube thoracotomy was performed immediately (first pneumothorax, Figure 2C). The pneumothorax resolved within two weeks, allowing for patient discharge. At this point, there was partial regression of HCC in response to the atezolizumab and bevacizumab regimen. Following an additional four cycles of the same treatment, the patient experienced dyspnea again and visited the ER. CT imaging detected a recurrent pneumothorax, necessitating another tube thoracotomy (second pneumothorax, Figure 2D). In July 2024, the patient developed a third pneumothorax (third pneumothorax, Figure 2E).

Pulmonary function tests (PFTs) indicated chronic obstructive pulmonary disease (COPD) with the following results: forced vital capacity (FVC) of 87%/94%, forced expiratory volume in one second (FEV1) of 98%/104%, FEV1/FVC of 72%/71%, and diffusing capacity for carbon monoxide (DLCO) of 73%.

Laboratory tests for autoimmune factors, including antinuclear antibody (ANA), antineutrophil cytoplasmic antibody (ANCA), rheumatoid factor (RF), anti-cyclic citrullinated peptide (anti-CCP), and anti-dsDNA, were all negative.

We performed a tube thoracotomy for the recurrent pneumothorax. Given the patient’s response to treatment, atezolizumab and bevacizumab chemotherapy was continued.

After the ninth cycle of atezolizumab and bevacizumab, a response evaluation CT revealed an increase in intrahepatic HCC. Consequently, the patient discontinued atezolizumab and bevacizumab treatment and has since remained stable, with no recurrence of the pneumothorax.

## 3. Discussion

Immune checkpoint inhibitors (ICIs) were first approved by the FDA in 2014, and as of November 2024, 11 types have been approved for use. Atezolizumab, used in our patient, was first approved in May 2016 [7]. Due to its relatively recent approval, reports of side effects, including pneumothorax, are ongoing, and this side effect remains rare. In this case, a pneumothorax—a relatively rare side effect—occurred. It frequently developed after the administration of ICIs but did not recur after their discontinuation, suggesting a strong correlation between ICIs and pneumothorax. As the use of ICIs continues to expand, we expect an increase in reports of associated side effects.

The mechanisms of IrAEs are not yet fully understood, but they are associated with the effects of ICIs and present as inflammatory or autoimmune side effects [8]. Some reports suggest a significant correlation between the occurrence of IrAEs and the therapeutic efficacy of ICIs [9]. The currently known mechanisms include T cell-mediated effects (such as increased T cell diversity, cross-reactivity between self and tumor antigens, and imbalances between T cell regulators and effector cells), B cell and antibody-mediated side effects, inflammation and cytokine-mediated side effects, and microbiome-mediated side effects [10]. Furthermore, recent studies using PD-1, PD-L1, and CTLA-4 knockout mouse models have elucidated connections and mechanisms related to specific diseases, although no studies have yet linked these mechanisms to pneumothorax [11].

We conducted a literature review of cases involving pneumothorax following similar immunotherapy and identified two papers with three cases (Table 1). In the first case, a 25-year-old female with metastatic osteosarcoma on atezolizumab experienced her third pneumonitis attack and a pneumothorax after the 35th cycle. The pneumothorax resolved spontaneously without intervention, and immunotherapy was permanently discontinued. In the second case, a 36-year-old female with renal cell carcinoma developed pneumonitis and a pneumothorax 2 months after starting nivolumab, requiring a tube thoracotomy. Immunotherapy was permanently discontinued [12]. In the third case, a 65-year-old male with small-cell lung cancer (SCLC) and underlying emphysema developed a pneumothorax 3 h after starting pembrolizumab. Despite this, due to a good response to anticancer treatment on restaging, immunotherapy was continued [13].

Based on the cases reported so far, a pneumothorax as an IrAE is very rare. Typically, when a pneumothorax occurs in the context of ICI treatment, it is associated with pneumonitis or secondary to mass rupture. However, in our patient, there were no signs of pneumonitis, no metastasis to the lungs, and no worsening of underlying emphysema, leading to recurrent episodes of a pneumothorax. Similarly, in the third case, a pneumothorax occurred without pneumonitis. While pneumonitis is listed in ICI side effect guidelines, there is no specific information about pneumothorax, requiring clinicians to make individual judgments. In the first two cases, treatment was discontinued due to a recurrent pneumothorax, whereas in the last case, the pneumothorax did not recur, and treatment was continued based on the positive treatment response.

Our patient required systemic therapy due to the recurrence of hepatocellular carcinoma (HCC) despite several local surgical treatments, transcatheter arterial chemoembolization (TACE), and radiotherapy. Current HCC treatment guidelines recommend a combination of atezolizumab and bevacizumab as the first-line therapy. A recent Phase 3 randomized controlled trial (RCT) demonstrated that the combination of durvalumab and tremelimumab was more effective than sorafenib in terms of response rate (20% vs. 6%) and overall survival (median: 16.4 months vs. 13.8 months) [14]. However, since durvalumab is a PD-L1 inhibitor like atezolizumab, it may carry similar side effects [15]. For patients contraindicated for immunotherapy, lenvatinib, sorafenib, or their combination is currently recommended [16]. In our case, due to the recurrence of a pneumothorax and disease progression, the treatment was switched to lenvatinib, which has a different mechanism of action [17].

To date, Phase 3 ICI trials have excluded individuals with comorbidities, resulting in insufficient data regarding ICI efficacy and safety for these patients. Known risk factors include age <60 years, a high body mass index, women on CTLA-4, men on PD-1/PD-L1 agents, and chronic smokers. Furthermore, underlying autoimmune diseases pose additional risks [18]. However, there is a possibility that ICIs will be used in the future, even in patients with autoimmune disease or immune-depressed patients, where it was contraindicated. In a study involving 112 autoimmune patients treated with ICIs, 79 patients (71%) experienced autoimmune flares and IrAEs, but most of them were manageable [19]. Additionally, another study indicates that combining ICIs with TNF or IL-6 inhibitors may mitigate serious side effects while achieving a decoupling effect between toxicity and efficacy [20]. A recent study involving 17 kidney transplant recipients treated with nivolumab for solid tumors reported no evidence of increased tumor progression, graft rejection, or IrAEs [21]. Given the high efficacy of ICIs, it may be preferable to continue treatment if side effects can be effectively managed.

Our patient had a 30 pack-year smoking history and was a current smoker. Recent studies indicate intriguing relationships between smoking and the efficacy of ICIs. For instance, a study involving 644 NSCLC patients showed that smoking was associated with improved clinical outcomes in ICI monotherapy [22]. Additionally, a systematic review found that smokers exhibited a higher overall response rate to ICIs [23]. This suggests that smoking may contribute to a higher tumor mutation burden (TMB), which correlates with immunotherapy response. Furthermore, the development IrAEs has been associated with positive outcomes from ICIs, indicating that these side effects may be linked to their effectiveness [24]. While specific reports on HCC patients like ours are lacking, it is reasonable to anticipate that smoking-induced stress could enhance ICI therapy’s efficacy while also leading to increased side effects.

In this patient, the VEGF inhibitor bevacizumab, used concurrently, could also be a potential cause of the pneumothorax. A systematic review of pneumothorax occurrences in patients treated with bevacizumab found that while this side effect is rare, five cases were reported (Table 2). Most cases involved ipsilateral lung lesions, but one case occurred without pulmonary metastasis [25,26]. Additionally, a study involving children and adolescents treated with sorafenib, bevacizumab, and cyclophosphamide found that pneumothorax, likely related to the therapy, developed in 11 out of 44 (25%) patients [27]. In another study, a pneumothorax was observed in 10.3% of 58 patients with soft tissue sarcoma (STS) receiving pazopanib [28]. Additionally, Kasahara et al. reported that VEGF inhibitors might induce cell apoptosis, leading to changes in the alveolar structure that could potentially cause pneumothorax [29]. However, a case–control study of pazopanib, which included 41 cases and 164 controls, concluded that pazopanib did not increase the risk of pneumothorax [30]. Overall, the association between VEGF inhibitors and pneumothorax remains unclear, and the possibility of pneumothorax related to ICIs cannot be ruled out.

## 4. Conclusions

When a pneumothorax occurs in a patient undergoing immunotherapy with ICIs, it should be considered a potential adverse effect of the treatment. It is especially important to assess the risk of exacerbating underlying lung disease due to immunotherapy. Before initiating ICIs in patients with a history of smoking or pre-existing lung conditions, a thorough lung evaluation—including a chest CT scan and PFTs—is advisable. Regular follow-up with chest X-rays is also recommended, and patients should be educated on the importance of smoking cessation. If dyspnea symptoms develop after ICI use in patients with existing lung disease risk factors, the possibility of pneumothorax should be promptly considered. Since there are currently no guidelines for resuming immunotherapy following the occurrence of a pneumothorax, it is essential to carefully weigh the risks and benefits and consider dose reduction or discontinuation of the treatment.

## Figures and Tables

**Table 1:** Literature review of pneumothorax as complication of immune checkpoint inhibitors.

Year	Country	Sex	Age	Diagnosis	Treatment	Onset	Prognosis	Ref.
2024	South Korea	M	80	HCC	Atezolizumab /bevacizumab	After 3 cycles of atezolizumab/bevacizumab	Immunotherapy continued	Our case
2021	Turkey	F	25	Metastatic osteosarcoma	Atezolizumab	After the 35th cycle of atezolizumab	Immunotherapy permanently stopped	Kucukarda A et al. [12]
2021	Turkey	F	36	RCC	Nivolumab	After 2 months from initiation of nivolumab	Immunotherapy permanently stopped	Kucukarda A et al. [12]
2020	Greece	M	65	SCLC	Pembrolizumab	After 3 h of pembrolizumab initiation	Immunotherapy continued	Sardeli C et al. [13]

Ref., references; M, male; F, female; HCC, hepatocellular carcinoma; RCC, renal cell carcinoma; SCLC, small-cell lung carcinoma.

**Table 2:** Literature review of pneumothorax as complication of VEGF inhibitors.

Year	Methodology	Case	Treatment	Prognosis	Ref.
2018	Literature review	6 case reports	Bevacizumab	In 5 cases, pulmonary metastases were present. However, there was 1 case of pneumothorax occurring after treatment without any lung lesions.	Alrifai T. et al. [25]
2022	Systematic review	5 case reports	Bevacizumab	Although very rare, pneumothorax can be a clinically significant side effect.	Rehman S. et al. [26]
2015	Case series	44 children and adolescents with refractory/recurrent solid tumors	Sorafenib, bevacizumab, cyclophosphamide	Pneumothorax developed in 11 out of 44 patients (25%). The formation of cavitary pulmonary nodules in response to therapy is a known risk factor for pneumothorax.	Interiano RB. et al. [27]
2016	Case series	58 STS patients	Pazopanib	The prevalence of pneumothorax was 10.3%, with an incidence of 0.56 per treatment-year. The median onset of pneumothorax occurred on day 115 (range: 6–311 days). Pazopanib was continued or restarted after 9 of the 13 events.	Nakano K. et al. [28]
2018	Retrospective case–control study	41 cases and 164 controls	Pazopanib	Pazopanib did not significantly increase the risk of pneumothorax in univariate (*p* = 0.06) or multivariable analysis (*p* = 0.342). In multivariate analysis, the presence of cavitary lung nodules or masses (*p* < 0.001) and pleural-based nodules or masses (*p* < 0.001) were the only significant risk factors for pneumothorax.	Sabath B. et al. [30]

Ref., references; STS, soft tissue sarcoma.

## Data Availability

The data are contained within the article.
